# Midwives’ care on a labour ward prior to the introduction of a midwifery model of care: a field of tension

**DOI:** 10.1080/17482631.2019.1593037

**Published:** 2019-03-29

**Authors:** Christina Nilsson, Olof Asta Olafsdottir, Ingela Lundgren, Marie Berg, Lisen Dellenborg

**Affiliations:** a Faculty of Caring Science, Work Life and Social Welfare, University of Borås, Borås, Sweden; b Sahlgrenska Academy, Institute of Health and Care Sciences, University of Gothenburg, Gothenburg, Sweden; c Department of Midwifery, School of Health Sciences, University of Iceland, Reykjavik, Iceland; d Obstetric Unit, Sahlgrenska University Hospital, Gothenburg, Sweden

**Keywords:** Midwifery, women, models of care, experiences, childbirth, ethnography, culture, woman-centred, work place

## Abstract

**Purpose: **There is a need to deepen knowledge about midwives’ care in obstetric-led labour wards in which midwives are responsible for normal births. This ethnographic study explores the content and meaning of midwives’ care of women in a hospital-based labour ward in Sweden prior to the introduction of a theoretical midwifery model of care. **Methods: **Data were gathered through participant observation, analysed through interpretation grounded in reflexivity discussions and are presented in the form of ethnographic descriptions. **Results: **The midwives’ care was provided in a field of tension in which they had to balance contrasting models of care, described in the themes: The birthing rooms and the office—Different rooms of care, Women giving birth or being delivered—Midwives’ expectations and relationships with women, Old and new caring roles of the midwife—Women giving birth in a “new age”, Being and doing—Different approaches to caring, and Holistic and reductionist care—Guided by contrasting models and guidelines. The midwives’ freedom to act as autonomous professionals was hindered by medical and institutional models of care and this led to uncertainty regarding their roles as midwives. **Conclusions: **Midwives having to balance their activities in a field of tension require midwifery models that can guide their practice.

## Introduction

Midwives’ care of women during childbirth is provided in a cultural and situational context. In high-income countries, childbirth care is primarily provided in hospital labour wards and the centralization of maternity services has created a culture of industrial models of care in which the institution constitutes a more significant social unit than the care of the individual or the family (Davis-Floyd, 1992, 2003; Newnham, McKellar, & Pincombe, 2017; Walsh, El-Nemer, & Downe, 2008). However, supportive care in childbearing is of great importance to ensuring positive experiences among women who give birth because such experiences can have a long-term effect (Leap & Hunter, 2016; Lundgren, Karlsdottir, & Bondas, 2009). Moreover, continuous support for birthing women is associated with more spontaneous vaginal births and fewer negative birth experiences (Bohren, Hofmeyr, Sakala, Fukuzawa, & Cuthbert, 2017). Reviews of continuity-based midwifery models of care compared to conventional models of care also report a higher rate of maternal satisfaction, fewer interventions and a trend towards a cost-saving effect (Sandall, Soltani, Gates, Shennan, & Devane, 2016).

Enabling meaningful relationships between midwives and women and their families is central in midwifery models of care (Hunter, Berg, Lundgren, Ólafsdóttir, & Kirkham, 2008; ICM, 2005). The presence of midwives at birth and the relationship between midwives and women has been described as having an effect on the development of midwifery skills, particularly the knowledge needed for promoting normality and safe births (Olafsdottir, 2006/2011). Globally, an evidence-informed framework for Quality Maternal and New-born Care (QMNC) has recently been developed (Renfrew et al., 2014). This framework comprizes health systems required by all childbearing women and their families and midwifery is considered a fundamental component of this framework (Renfrew et al., 2014). Various studies into midwives’ practice show that midwives feel that their work takes place between different “belief systems”(Blaaka & Schauer Eri, 2008), based on “conflicting models of care” (Olafsdottir, 2006/2011), in which the midwives’ approaches vary between being “with the woman” or being “with the institution” (Hunter, 2004) in workplace cultures in which they feel they are being monitored, as well as being controlled by clinical guidelines (Davis & Homer, 2016).

In a Nordic context, a theory-based woman-centred Midwifery Model (MiMo) of care in childbirth has been developed based on qualitative research findings about women’s and midwives’ experiences of childbirth in Swedish and Icelandic settings (Berg, Ólafsdóttir, & Lundgren, 2012). Three central intertwined themes of the MiMo describe how midwives form a reciprocal relationship, create a birthing atmosphere and use their grounded knowledge to provide woman-centred care. In the background are components that influence how these central aspects of care are performed, including the cultural context with its hindering and promoting norms, as well as the balancing act in the practice of facilitating effective care for a woman and her family (Berg et al., 2012). Further evaluation of the use of the model is necessary, including its usefulness and impact on midwifery practice in labour wards. However, a better understanding of midwives’ care is needed before such a model can be introduced. The aim of this study was to explore the content and meaning of midwives’ care of women in a hospital-based labour ward in Sweden with an expected normal birth prior to the introduction of a theoretical midwifery model of care: the MiMo.

## Methods

This study forms part of the MiMo research project. The research project had a mixed method research design, as described by Morse (2009), with the overall aim of exploring the usefulness of the MiMo and its impact on the outcome of childbirth care. This study drew on ethnographic methods that were used to explore the content and meaning of midwives’ care during childbirth before the MiMo model was introduced in a hospital-based labour ward. Data were collected during January and February 2015.

Ethnography has been established as a method of understanding healthcare contexts (Long, Hunter, & van der Geest, 2008; Roper & Shapira, 2000). This method is appropriate for the aim of this study and was inspired by Leslie, Paradis, Gropper, Reeves, and Kitto (2014) and Skott, Dellenborg, Lepp, and Nässén (2013). Ethnography aims to understand human behaviour and culture from a local point of view and through devoted descriptions and critical reflections it aims to gain insights into the “ways of living of others and their interpretations of the world” (Skott, 2013a, p. 52). Participant observation was used and this constitutes the main ethnographic method (Nässén, 2013), with its main strategy comprising fieldwork conducted over a period of time (Dellenborg, 2013). The aim of the observations was to keep an open mind in order to understand organizational and cultural behaviours from within (Borneman & Hammoudi, 2009).

Hospitals are complex organizations characterized by caring for people in great need, as well as by different professional groups, disciplines and knowledge that are often hierarchically related (Erichsen Andersson et al., 2018; Wolf, Ekman, & Dellenborg, 2012). Ethnography is a research method that is beneficial to use when there is limited knowledge of the research subject, as well as its culture and its interpretations (Long et al., 2008). This applies to the study presented here: the content and meaning of midwives’ care of women in a hospital-based labour ward in Sweden with an expected normal birth prior to the introduction of a theoretical midwifery model of care: the MiMo.

### Setting, the studied labour ward

This study was conducted in a labour ward for normal labour and birth at a hospital in one of the largest cities in Western Sweden. The maternity organization incorporated two additional labour wards, one for special obstetrics (women with complicated pregnancy, labour and birth) and, in another building, one for normal obstetrics. In 2015 the hospital had 10,050 births, 17.1% of which were by CS (Caesarean Section) and 4.2% of which were instrumental vaginal births (vacuum extraction). The actual labour ward had 4556 births in 2015 and mainly focused on normal obstetrics, i.e., women with singleton uncomplicated pregnancies and expected uncomplicated births from gestational weeks 37–42. However, induction of labour was common, as well as women with minor complications during pregnancy such as gestational diabetes and gestational hypertension. Women with expected uncomplicated births from gestational week 34 and women with stillbirths were also cared for in the ward. Women at high risk gave birth in the same building in a special ward for women with complicated pregnancy, labour and birth. Approximately 80 midwives were employed in the ward and worked three shifts—day (06.45–16.00), evening (14.00–22.00) and night (21.00–07.00). In the studied labour ward it was common for midwives to have to care for more than one woman in labour; one-to-one care for all women was not possible. Continuity of care is not offered in the actual ward because the care during pregnancy is provided by other midwives employed in primary health care.

In Sweden, midwives in hospital labour wards have an independent role with responsibility for women having normal pregnancies, labour and birth. If complications arise, physicians assume responsibility, but the midwives remain involved in the woman’s care. Maternity care is funded by the state and is free of charge. From an international perspective, the rates of maternal and perinatal morbidity and mortality in Sweden are low. In 2015, the national rate of CS in Sweden was 17.4% and in 2016 it was 17.6%. However, the rate varies across regions from 12.1% to 21.8% (2015) and 12.3% to 21.6 in 2016 (National Board of Health and Welfare, 2017, 2018).

### Data collection and participants

During January and February 2015, ethnographic fieldwork (Borneman & Hammoudi, 2009; Dellenborg, 2013) and participant observation (Nässén, 2013; Savage, 2000) were conducted by the first author (CN) in which six midwives were followed during their day, evening and night shifts. Field notes were taken during the observations and informal talks and brief interviews were conducted with the midwives. The data also comprized the researcher’s reflection notes and in-depth interviews with two of the observed midwives, as well as additional in-depth interviews with a further two midwives. All data were collected by the first author (CN). Being both a researcher and a midwife conducting fieldwork in a labour ward required methodological reflections in order to avoid bias in the interpretations (Roper & Shapira, 2000), which are further described in the section on the study’s strengths and limitations.

The six observed midwives who took part in the study were between 27 and 63 years of age and had 1 to 31 years’ working experience on labour wards. The two additional midwives who participated through interviews only were 35 and 45 years of age. One had two years’ work experience and the other had 16 years’ work experience on the labour ward. They volunteered to participate when the researcher asked for other midwives to take part in in-depth interviews about the care of women during labour. The reason for performing these interviews was to expand our understanding of our tentative interpretations of the observations by asking some key questions, for instance: “How do you perceive your professional care and support for women?”. The interviews were conducted in a spare room at the labour ward normally used for meetings and lasted approximately 45 minutes. The interviews were recorded.

The midwives were selected by the senior midwife, who coordinated the daily work at the labour ward, mainly based on the relevant midwife caring for a woman in active labour (first or second stage of labour). Variation in length of working experience and age of the participating midwives was also desired when selecting participants. After being invited by the senior midwife, the midwives received brief information about the study. If they expressed an interest in participating, they were approached by CN, who provided more detailed information, both verbally and in writing. None of the selected midwives chose not to participate. The midwife, in turn, gave the birthing woman (and her partner) initial information about the study. If the woman (and her partner) were interested, they were approached by CN. They received detailed information verbally and in writing and had the opportunity to ask questions. The women were informed that they were not the focus of the observations; it was the midwives’ care that was being studied. This conversation also gave the women and the researcher the opportunity to become acquainted. One of the women’s partners reacted negatively to having a researcher in the birthing room, resulting in the woman declining to contribute to the study. None of the other five women who were approached declined to be involved in the study.

### Data analysis

The methodology of ethnography includes reflexivity, description, comparison and interpretation, with an inner perspective and a relativistic stance (Scott-Jones & Watt, 2010; Skott, 2013b). In ethnography, descriptions and interpretations are acknowledged as being inseparable, and a hermeneutic approach is essential (Skott, 2013b); the researcher translates and interprets while he or she is observing, and the analytical process begins as soon as the researcher starts taking field notes. Crucial to the reliability of an ethnographic study is the ethnographer’s reflexive stance that aims to gain an awareness of his/her own pre-understandings. An ethnographic understanding is attempted by making detailed descriptions of social milieus and people’s experiences, perceptions and practices in context, a so-called “thick description” (Geertz, 1973), which “does more than record surface appearances” (Leslie et al., 2014, p. 100).

The handwritten field notes and reflection notes were transcribed by CN and the recorded interviews were transcribed verbatim. Before, during and after the fieldwork, CN (a midwife and researcher experienced in qualitative methods), the last author (LD, an experienced anthropologist), and author OAO (a midwife with experience of ethnographic research) conducted numerous research meetings to discuss, analyse and further interpret CN’s primary interpretations. The focus of the observations and the interviews was the content and meaning of the midwives’ care in the specific context. The analytic process started during participant observations by taking field notes, engaging in informal talks, conducting interviews and later, taking reflection notes. The analysis followed the hermeneutic spiral method (Gadamer, 1995/1960) in order to understand the midwives’ care and experiences, conceptions, perceptions and practices (Skott, 2013b). Ethnography is a hermeneutic enterprise, which entails going from the whole to the parts and back again, repeatedly, constantly reflecting on one’s own preunderstandings (Roper & Shapira, 2000). This process of understanding was conducted by formulating tentative interpretations, leading to the framing of new questions and interpretations. Tentative themes, with their contents and meanings of the midwives’ care, were formulated and reformulated. In this way, different interpretations were tested and mirrored between each other through numerous discussions in the research team. This process of analysis resulted in the five themes presented below, together with ethnographic descriptions of the content and meaning of the midwives’ care in the specific context. The regular research team meetings involved collective work by reading tentative descriptions and interpretations and giving feedback, while simultaneously focusing on understanding the midwives’ inner perspective as well as the context of their care. Vital parts of the meetings comprized the researchers’ reflections and comparisons of their preunderstandings, as well as formulating and questioning the emerging interpretations. The analysis was further revised and verified by the other authors (IL and MB).

### Ethical approval and considerations

Ethical approval for the study was obtained from the Regional Ethical Review Board in Gothenburg (ref. no. 840–14). Permission to conduct participant observation during childbirth and to audio record the interviews was received in writing from each participating midwife, as well as the women in their care. The participating midwives and the women signed consent forms and were guaranteed both verbally and in writing that all information would be treated confidentially and that they could withdraw their participation whenever they wished. Additionally, the women were assured that their participation, or non-participation, would not impact their care.

## Findings and reflections

The midwives’ care is described and reflected in five themes presented in the form of ethnographic descriptions. When analysing the data, it became evident that the midwives worked at the labour ward in a field of tension. This field of tension was characterized by the midwives having to balance contrasting models of childbirth care with ambivalent relationships between midwifery, medicine, institutional care and hospital organization. The field of tension was further exacerbated by the midwives’ physical movements between different rooms in the labour ward, as well as their practice in the various rooms. These rooms can be seen as representing the various standpoints on how care should be performed and what a midwife’s role is in childbirth care, as well as the various understandings of what midwifery really is about. Thus, the word “room*”* is used here in a metaphorical, physical and existential sense.

The themes that illustrate different aspects of the midwives’ constant movement between these rooms, their practice, and the different standpoints on childbirth care are:

1. The birthing rooms and the office: Different rooms of care
2. Women giving birth or being delivered: Midwives’ expectations and relationships with women
3. Old and new caring roles of the midwife: Women giving birth in a “new age”
4. Being and doing: Different approaches to caring
5. Holistic and reductionist care: Guided by contrasting models and guidelines

### The birthing room and the office: Different rooms of care

The midwives constantly moved between the birthing rooms and the office, rooms in which different kinds of care took place. In the birthing rooms the midwives took care of individual women, in contrast to the office, from where the care for the women was governed and monitored. When the labour ward was busy, they described how they had to move in and out of the birthing rooms and address the women’s various needs. The midwives were worried about not being able to manage their tasks because of their heavy workload. One midwife said she sometimes worried about becoming jaded from overwork and being too tired to engage with all of the birthing women. The heavy workload could perhaps be one explanation of the observed contradiction whereby when they were in the birthing rooms, the midwives appeared to be drawn to the activities in the office, and vice versa: when they were in the office they were drawn to the women in the birthing rooms.

Activities outside the birthing rooms were often extensive but did not necessarily mirror the activity inside the rooms. When opening the door to one of the birthing rooms, the whole room, including the woman and her partner, was immediately on display. All birthing rooms were designed and equipped in the same way with a birthing bed positioned in the centre of the room surrounded by technical equipment. Alongside one of the walls were cabinets filled with equipment, with labels placed on each door describing the cabinet’s contents. The atmosphere in the birthing rooms varied. One midwife emphasized the importance of sensing the mood in the room and approaching the woman and her partner based on that sense. She described how she approached the rooms in terms of coming home to different people, all with different personalities and bringing different things to the room. For examples of two different birthing rooms, see Text box 1.

**Text box 1.** Vignette: A look into two of the birthing rooms.

The atmosphere in the birthing room during one observation was very intense, but still calm and filled with trust. The birthing woman was sitting like a queen in her bed; she had strong contractions and inhaled Entonox with silent breaths. Her mother and sister were there, holding her hands. The midwife was present at her side, occasionally listening to the baby’s heart by using a Pinard. She later described the ward as being very hectic that day, but in this room it was calm. When the researcher informed the woman that the study concerned midwives’ care, she said with a big smile that her midwife was wonderful: “You must pass her with distinction. She was breathing with me, and that made me calm”. ***** In another room, a multiparous woman had very painful contractions. The situation seemed rather chaotic, but the young midwife was calm and appeared to be in control. The woman lay in the bed, connected to a CTG monitor through a wire, and inhaled Entonox heavily. She screamed occasionally, but listened to the midwife. Her husband was at her side holding her hand, looking worried. The midwife had a plastic smock on and all the birthing utensils ready at a table. She urged the woman to try to relax and listen to her. While observing the CTG and the woman’s genitals, she asked the auxiliary nurse to call for the assisting midwife to hand her oxytocin. After a short while the baby’s head crowned. The midwife held her hands on the baby’s head and the woman’s perineum, and her face turned slightly red because of the effort. The baby was born, but didn’t cry immediately. The midwife put her hand on the baby’s chest and said everything was fine. She wiped the baby dry, soon he cried and the mother and father looked very happy. Within the next few minutes the midwife had taken care of the following duties: she took a blood test from the umbilical cord before cutting it, placed the baby onto the mother’s chest, checked the woman’s uterus by placing a hand on the abdomen, gave an injection with oxytocin, helped the placenta out, checked that the placenta was complete, and thereafter examined the woman’s genitals for possible tears. The midwife was focused and worked very quickly, but respectfully by sometimes apologising for disturbing the woman.

In contrast to the smaller birthing rooms, the office comprized one large room—the labour ward’s “nerve centre” and a meeting point for the staff. Nevertheless, the birthing women were still present in the form of numbers and names on a whiteboard with information on their actual status, including a box for risk assessment in which the birthing women were assessed as being low, medium, or high risk. Many activities important for the women in the birthing rooms were controlled from the office. All rounds and most discussions and decisions took place in the office. During one of the regular morning rounds, the office was crowded and the sound level was high. When the head obstetrician arrived together with three other physicians, the round started and everyone became quiet. The room was hot and the atmosphere felt strained. About 10 midwives, together with a few midwifery students, briefly presented their cases. Some of them used the SBAR communication technique (Situation, Background, Assessment and Recommendation), the recommended tool for communication between professionals on a ward. The focus was solely on the medical aspects of childbirth. All staff listened and after each case the head obstetrician posed some questions to the midwives and gave some concluding recommendations, as well as some prescriptions, if necessary.

The office contained several workplaces with computers and phones, as well as two sofas and a table. The office seemed to attract the midwives. They often had their coffee break there and, when the ward was short-staffed, also their lunch and dinner. The office’s role of serving as a breathing space was necessary for managing all of the hours spent in the birthing rooms; they could relax here for a while. The midwives met each other in this room; they got to know each other and chatted, they shared birth stories, experiences and knowledge, and expressed their feelings and frustrations. The office contained all kinds of feelings—stress, irritation, strain, anxiety, fear, relaxation, friendship and joy.

At the same time, the midwives always kept an eye on the numerous monitors with CTG (cardiotocography) and STAN (ST waveform analysis) registrations, which were suspended from the wall, dominating the office. The monitors were connected to each birthing room via a combined CTG and STAN monitor placed beside the bed for monitoring of the baby’s heart and the woman’s contractions. The screen was connected to the office and supervized from there. This monitoring could be a source of anxiety and irritation between midwives and physicians, as described below. Through the CTG monitors, the activities in the birthing rooms were, to a certain extent, redirected to the office and were transparent to all staff. Consequently, activities in the birthing rooms could be controlled from the office. However, this transparency was limited to some extent and the CTG registration did not describe everything that was going on. The midwife inside the room had total knowledge of what was going on, while the other staff in the office could only observe the CTG without having the entire picture of the birth process; they were, to some extent, both inside and outside the birthing rooms. This caused anxiety and irritation among the midwives, particularly when senior midwives or physicians interacted by knocking on the door to the birthing room to check with the midwife about what was happening. Such circumstances created uncertainty, particularly if there was no consensus on how to interpret the CTG pattern. One experienced midwife explained how frustrated she was at the feeling of being controlled by inexperienced physicians in the office whom she considered felt safer with caesarean section than with vaginal birth. She avoided the office and preferred to be with the women in the birthing rooms. Midwives in the birthing rooms were very much aware of this transparency; they were, to some extent, under surveillance, which caused tensions between the different rooms, between the midwives, and between the midwives and the physicians.

### Women giving birth or being delivered: Midwives’ expectations and relationships with women

The midwives experienced that women had different approaches to childbirth; some women wanted to be delivered, while other women wanted to give birth. They described birthing women as being different; not all women want natural births, which indicates a conflict between the wishes of midwives and birthing women. The midwives preferred women who wanted to give birth naturally but unfortunately this did not happen often. Caring for a woman who gave birth naturally was described as “real midwifery”. Normal physiological births were quite rare and they thought it was unfortunate that epidurals were so strong, given the knowledge that women’s bodies work so well by themselves. As one midwife said: “It’s so nice to see a primipara who doesn’t demand an epidural”.

The midwives’ goal was to make the women feel as if they were giving birth by themselves. This included helping anxious and insecure women overcome their fears and believe in themselves, which could also serve as confirmation of their midwifery skills. However, the midwives admitted that it was often difficult to read birthing women who were in intense pain. However, they searched for the key to each woman and tried to understand her as a unique person, even though this was sometimes challenging. It was important not to give up and one midwife stated how she always tried to be one step ahead so that a woman really felt that she cared for her. The midwives thought it was easier to establish a caring relationship when they felt sympathy and warmth for a woman and were acknowledged by her.

When the midwives failed to establish a good relationship with a woman, they had to accept it. One common obstacle was language and the midwives stated that it was harder to communicate with a woman who could not speak Swedish. Such a situation was also described as an interesting challenge and the midwives had to be creative in their efforts to find other ways of communicating. They also experienced that women are different and some women did not need them as much as others.

### Old and new caring roles: Women giving birth in a new age

The midwives stated that a new generation of birthing women who requested service on a 24/7 basis had emerged. One midwife reflected on the phrase “the McDonalds generation”, which she meant as a label to describe a generation of women who are used to having their demands met immediately. The midwives considered that epidurals were stronger and more effective than previously and they described their caring role as being different when the birthing woman had received an epidural. It was not unusual for a woman, despite being in an advanced stage of labour, to be completely pain-free except for a sense of pressure in the back. The midwives described such women as being in almost a normal state, not in “birthing mode”. Generally, women in an advanced stage of labour were described as introverted and focused. They were affected by the pain and greatly in need of the midwives’ support. In contrast, women who had received an epidural may have spoken with their friends on the phone, have eaten large meals, slept, watched films, cuddled their partners and checked their Facebook status. The midwives stated that their supporting role was diminished; they were not needed and felt it was sometimes hard to justify their presence in the room. Some women, as well as some midwives, appeared to feel more secure when using medical pain relief, CTG monitoring and medical interventions. The women wanted painless births and, because of being administered heavy pain killers, they were not entirely dependent on the midwives’ support. The independence of such women in relation to the midwives, at least during the first stage of labour, required a new and different caring role for the midwives.

Consequently, some women have demanded another kind of midwifery. In relation to such women, the midwife’s role has changed and he/she has become more of a controller who checks the progress of labour and the baby’s condition than a supportive midwife who spends time with a woman. Interaction with such women is more shallow and one midwife described the conversations as being more neutral, like having a conversation with a neighbour. The midwives felt that the strong effect of the epidurals seriously disrupted a woman’s birthing focus and took away her bodily connection. It was clear to the midwives that they were not needed as much during the first stage of labour, although they were still needed during the second stage when the women needed their help to eventually give birth. Many midwives preferred women who wanted natural births and were happy when they could attend such births and perform their traditional caring role. Sometimes their heavy workloads made the midwives question their ability to remain in their traditional supporting role (see Text box 2). The midwives felt unsure, questioning their role as a midwife in relation to the birthing women’s needs and to their workload.

**Text box 2.** Vignette: Epidural.

One day, the following was observed at the labour ward: A woman in advanced labour who was recently admitted told her midwife that she wanted an epidural. The midwife asked her to reconsider and explained that the preparations would take much of her time, with the risk of leaving the woman alone without a midwife being at her side. Despite the midwife’s concerns, the woman chose the epidural. However, when all the preparations were completed she started to push and gave birth just a few minutes later. The midwife explained later to the ethnographer that she wanted to be honest with the woman and make sure she understood the possible consequences of her choice. The midwife’s interpretation was that the woman did not dare to give birth without the pain relief, while from her experience she knew that she as a midwife had the possibility to support and induce courage with her presence.

### Being and doing: Different approaches to caring

The different birthing approaches among the women described above (depending on the women’s choices and/or the midwives’ workload) were mirrored by midwives who had different approaches to caring. These approaches could be called “the being approach” and “the doing approach”. Individual midwives used aspects of both approaches and moved between them depending on the situation. However, observations showed that some of them focused more on the being approach than on the doing approach, and vice versa. The different attitudes created tensions within and between the individual midwives.

*The being approach *entailed focusing on the birthing women’s genuine needs, rather than their personal wishes. This approach also entailed a state of being that radiated confidence and authenticity; the midwives filled the room with their presence and confidence. As one midwife explained, it is important to have a personal style as a midwife, based on who you are as a person. Moreover, ther being approach saw the whole picture, even the smaller details. They collected small pieces of information and continuously added to their knowledge, each without losing sight of the bigger picture of the birthing woman.

For instance, this approach entailed not offering any suggestions regarding pain relief. Instead, the midwives observed and waited for the woman to make such a request. If the midwife believed that the woman could give birth better without an epidural, she would say so. However, an epidural could be recommended by the midwives to a woman who really needed one. The being approach also entailed not making a big issue out of having to use the CTG, the monitor was not in focus. A Pinard stethoscope or a doptone were often used when monitoring the baby. CTG technology was used as an aid, but only when necessary.

The being approach entailed watching over the woman; the midwives observed, felt and followed the woman and confirmed to her that they were doing so. One midwife told the ethnographer how she had observed one woman who moved her legs in a particular way during contractions and, based on this observation, she knew that the second stage of labour was imminent. This approach also entailed supporting women on their own terms; to help them stay on track during birth. As one midwife said: “Actually, most women know how to give birth, you only have to reassure them in what they are doing”. The midwives’ being approach also included an attempt at connecting with the women. This kind of relationship was more than just about making positive contact, it was about being connected, being in sync with each other. While the woman was reassured in her birthing, the midwife received an acknowledgement that her care had really supported the woman and that she had understood the labour process correctly. One midwife was observed making gentle murmuring sounds when she sat beside a woman who was in intense labour. She said that she produced this sound naturally and explained that it was a way of reassuring the woman that she was with her.

*The doing approach* represented a kind of caring that included doing things for the birthing women and, in contrast to the being approach, the midwives were fully occupied with tasks such as check-ups relating to foetal and maternal measures, administration and documentation of the patient records. Although a midwife might be concerned about a woman’s specific request, her doing approach tended to imply a rather non-problematizing attitude to a women’s wishes and more of a service-minded attitude to a birthing woman’s requests. In contrast, midwives who adopted a being approach sought to understand what a woman’s deeper needs might be, for instance, to be supported when she asked for pain relief. The doing approach also signified constantly doing things, for instance, constantly talking to a woman, even at the risk of disturbing her. It appeared that in such instances, the midwives found it difficult to just remain silently with a woman, trying to connect with her. However, doing things could also be used to justify “only being” with a woman. For example, in one observation it was obvious that during a penicillin injection that had to be administered very slowly the midwife was given the opportunity to really interact with the woman. This interaction resulted in a strengthened relationship, which was later acknowledged by the midwife.

Generally, the doing approach implied more instances of actively offering a women pain relief. Although some women did not ask for it, offering pain relief appeared to be an important task, in which the women were subtly asked: “Are you still OK?”. For instance, despite a woman being hesitant about using Entonox, one midwife firmly asked her to at least try Entonox, rather than support her desire not to use it. The doing approach also implied a different attitude to the use of the CTG. Besides monitoring the baby, the CTG was also used as a tool to help women control their contractions. For instance, instead of terminating a CTG registration that had been completed, one midwife asked a woman if she wanted to continue the monitoring to in order to help see when the contractions appeared, even though the woman could feel them. The midwife explained to her that it was easier to match the contractions with the Entonox in this way: “Actually, we don’t need the CTG for the baby’s sake—its heart rate is fine”.

The doing approach appeared to be more focused on tasks than the being approach, which implied a more woman-centred attitude. These dissimilar approaches created tensions among the midwives, leading to different perspectives on what the right kind of midwifery was. For instance, when changing shifts a midwife was relieved when she discovered that the next midwife was “the right kind”, using a more being approach without disturbing the woman. However, their heavy workloads required each midwife to care for many women and they had a lot of duties and tasks, sometimes forcing them into adopting a doing approach they did not want to adopt.

### Holistic and reductionist care: Guided by contrasting models and guidelines

As noted, the midwives were guided in their care by contrasting models and guidelines, which created tensions between midwives and physicians, as well as tensions with the directives of the institution and within the group of midwives. Generally, the midwives adopted a holistic perspective that emphasized wholeness and the normality of birth, in contrast to the reductionist medical and institutional models of care that primarily focus on the pathological perspectives of childbirth.

Tensions appeared in relation to the physicians’ medical model of care, as well as the institutional demands regarding efficiency and bureaucracy that were often articulated in the local guidelines that all staff had to observe. However, tensions also existed within the group of midwives, with some of them having adopted more of a reductionist view on birthing women. The prevailing question for the midwives, either outspoken or subtle, was whose understandings were the most valid. Were the midwives permitted to work according to the model of holistic care or should they work according to the model of reductionist care? See Text box 3 for an additional example of tensions between different models of care.

**Text box 3.** Vignette: Examples of tensions between different models of care.

A midwife and a physician had a discussion in the office concerning which method to use for inducing a woman’s labour. The midwife suggested a method having the drug already prepared in her hand. The physician instead pondered on different suggestions from colleague physicians. The discussion ended with the physician saying: “I will examine the patient and discuss the case with my colleague, and then I let you know what to do”. Despite the determined tone, the physician seemed unsure about the midwife’s suggestion and wanted to consult with a colleague, excluding the midwife from this talk. The midwives in turn tended to exclude physicians because they preferred to consult with each other before consulting the physicians. They felt that the morning rounds with physicians focused on the medical and organizational aspects of childbirth to the exclusion of midwives’ views on the normality of childbirth and caring issues. The midwives therefore discussed the need for a midwife-round before the ordinary round with the physicians where they could consider more midwifery caring issues and not just the medical and institutional aspects of childbirth as they usually did during the morning rounds.

Moreover, the midwives expressed the opinion that newly-graduated midwives tended to have a more pathological view of childbirth. One of the experienced midwives explained that when supervising midwifery students she felt a strong urge to help them learn to trust the idea that women can actually give birth without interventions because many of the students were often inclined towards intervention.

Midwives and physicians often referred to the guidelines when they discussed women’s care. Frequently used phrases included: “Have you checked the guidelines” or “According to the guidelines we should…”. There were many guidelines to remember and many forms to complete. When disagreeing with the physicians, the midwives were sometimes outspoken in their opinions, were sometimes more cautious about speaking, or did not articulate their concerns at all. The medical model appeared to predominate with the physicians having the preferential right of interpretation and decision making.

The guidelines for the interpretation of CTG monitoring gave recommendations on how to interpret and act on the various patterns. However, the recommendations were not always in line with evidence-based practice and this caused frustration among the midwives, as one stated: “This feels strange because there is no scientific evidence for the use of admission CTG during normal labour”.

Moreover, it appeared as if certain controls were only for the records, not for the benefit of the woman and her baby. As an example, one midwife expressed relief when she discovered, having checked the guidelines on the CTG, that she did not have to disturb a woman taking a bath for the next two hours in order to do a CTG test. Another midwife was happy that a woman, whose labour was induced, “received two additional hours” before she had to start oxytocin infusion. Furthermore, midwives were happy about the guidelines for active labour regarding checking the baby’s heart rate every 15 minutes instead of using the CTG, even though they admitted that when they were busy they used the CTG because checking the CTG on the monitors in the office rather than in the birthing room with the woman was less time-consuming.

There appeared to be a conflict between the midwives’ model of care and the guidelines that often mirrored the medical and institutional views of childbirth. The midwives’ responsibility to comply with the guidelines was sometimes in conflict with how, according to their own knowledge, they perceived an individual woman’s physical and emotional needs. Some midwives were not always guided by their observations, experience and knowledge when caring for a birthing woman. Instead, they felt all they had to do was comply with the guidelines. When they sometimes deviated from the guidelines, even though they believed they were doing right, they felt uncomfortable and were afraid of doing something wrong, as well as being unsure about which view was valid. However, some midwives allowed themselves to ignore the guidelines, as exemplified by the midwife in Text box 4. Based on her professional knowledge and extensive experience, this midwife developed knowledge of the actual women and allowed this knowledge to guide her care, instead of the guidelines.

**Text box 4.** Vignette: In labour or not?.

One midwife with long experience told the ethnographer how she collects information from the women. When a woman is admitted, she not only asks about her obstetrical data, but she also wants to know about the woman´s whole life. This included her mother’s and other family member’s childbirths. She listens to each woman’s individual story, collects information, and then uses this knowledge in her care. This midwife was observed when she cared for a woman having no obvious signs of labour; she had no contractions and the previous cervix status showed only a minor opening. According to the guidelines, the woman should be sent home. However, this did not concern the midwife, and after she had made her examination and observations she concluded: “This will be a good birth, the woman has the right attitude; she wants a normal birth and her mother also wanted a vaginal birth when she gave birth to her daughter. She must stay”. One hour later the woman had stronger contractions.

Moreover, some midwifery skills were invisible in the guidelines. One midwife explained her strategy for how she helped some women with what she called “heavy” epidurals eventually give birth. These mobile epidurals are very effective in relieving pain, to the extent that women appear to be unaffected by their contractions. Because some women who have received an epidural cannot feel their contractions and the birth taking place in their bodies, they need help to realize it. In addition to what she usually did during the second stage, the midwife also encouraged the woman to concentrate on the birth by talking about her baby. She used the woman’s sense of pressure towards her back to make the woman more aware of her contractions.

In summary, the medical and institutional models of care permeated the atmosphere in the labour ward. The midwives’ knowledge of normal childbirth and of the unique women in their care was not seen as sufficient and was also made invisible. Instead, care for the birthing women was regulated by medical and bureaucratical models. This created uncertainty within and between the midwives and affected their collaboration with the physicians.

## Discussion

This study explores the content and meaning of midwives’ care of birthing women in a hospital-based labour ward in Sweden with an expected normal birth prior to the introduction of a theoretical midwifery model of care. The main findings describe how the midwives perform care in a field of tension in which they have to balance contrasting models of care. The midwives moved constantly between different rooms and perspectives of how childbirth care should be performed, with an ambivalent relationship between midwifery, medicine and the institutional organization. These circumstances obstructed the midwives’ freedom to act as autonomous professionals and created doubt and uncertainty regarding their own knowledge, regarding their caring role, and whether they were aware of the genuine needs of a woman during childbirth. These findings demonstrate the significant impact of the workplace culture on midwives’ care of birthing women, particularly in birthing cultures dominated by reductionist paradigms such as medical and institutional models of care.

The results presented here are in line with other findings about opposing models that guide childbirth care in hospitals (Blaaka & Schauer Eri, 2008; Davis & Homer, 2016; Hunter, 2004; Olafsdottir, 2006/2011). Being in this field of tension can also be related to the different perspectives and the dominance of the medical/technocratic vs. social/midwifery models of childbirth care (Bryar & Sinclair, 2011; Davis-Floyd, 2003; Hunter, 2006). When women’s care during labour and birth became gradually dominated by medical perspectives, the female birthing body came to be regarded as “a machine, with the doctor seen as manager, the woman as labourer and the baby as the product” (Martin, 2001, p. 64). The question is about how midwives in contemporary birthing care are seen and affected in a hospital context. The findings from a recent review of how perceptions of risk impact on professionals working in hospitals demonstrate that a focus on risk tends to increase the number of interventions when caring for women, even when the perceived risk is low (Healy, Humphreys, & Kennedy, 2016). In an ethnographic study of the culture in a larger hospital labour ward in Australia (Newnham et al., 2017; Newnham, McKellar, & Pincombe, 2018), a contradiction between risk and safety was revealed as “the paradox of the institution”. In the efforts to keep birthing women safe, their birth physiology and individual needs were sometimes neglected by midwives and physicians, which could put the women at risk (Newnham et al., 2018). Moreover, midwives’ professional support tends to be perceived as being vague in relation to the medical controls in detecting risks (Hunter, 2004; Thorstensson, Ekström, Lundgren, & Hertfelt Wahn, 2012; Walsh et al., 2008). Importantly, it seems that midwives’ professional identity in this process is weakened or even obscured (Healy, Humphreys, & Kennedy, 2017). This is reflected in our study by the midwives who felt uncertain about their roles and their knowledge when constantly having to balance conflicting models of care, with the potential consequences of the midwives feeling undervalued. Studies show that this leads to midwives tending to feel powerless and resigned in relation to the birthing women and their working situation (Catling, Reid, & Hunter, 2017; Healy et al., 2017).

Interestingly, our results indicate that working in a medically-dominated context can also imply the development of new roles and knowledge for the midwives. When the birthing women in our study received effective pain relief such as epidurals, they were described by the midwives as seeming less dependent on them, perhaps not needing to be at their side during the birth process. This could be problematic for some midwives who stated that they experienced difficulties when attempting to be with women who perhaps did not want to be with them. This is in accordance with the findings of Newnham et al. (2017) about how the impact of technology does not simply influence midwives but alters the environment in which care is provided, and ultimately the needs of the women. The midwives need to redefine not only their professional role, but also develop new knowledge, which has been observed and exemplified in our study in relation to women who had received an epidural. In order to avoid new knowledge being tacit, we need to conduct research in order to explore and verbalize such new understanding and to add this to midwifery knowledge. Recent studies on tacit knowledge of midwives’ care when working in health-oriented birth settings (i.e., normal birth settings in hospitals, birth centres and homebirths) clearly demonstrate how midwives’ praxis links to the concept of Sense Of Coherence (SOC) and salutogenesis (Magistretti, Downe, Lindstrøm, Berg, & Schwarz, 2016). This raises the question of whether the midwives in our study who experience contrasting models of care (Blaaka & Schauer Eri, 2008; Bryar & Sinclair, 2011; Hunter, 2004; Olafsdottir, 2006/2011) are in a field of tension because they experience obstacles towards salutogenic views and a promotion of pathology, thus preventing them from working as autonomous professionals based on their own knowledge. These are interesting findings, particularly when taking into account that midwife-led continuity models of care for healthy, low-risk women have shown indisputable benefits for women with regard to fewer interventions, higher levels of satisfaction and reduced costs with no differences in woman or infant outcomes when compared to other care forms such as medical-led models of care (Sandall et al., 2016).

The midwives in our study wanted to care for women undergoing normal physiological labour and birth without any interventions. They considered such care to be “real midwifery” and was described in a metasynthesis of midwifery care (O’Connell & Downe, 2009) as an overly “idealized” approach that is difficult to attain in hospital labour ward settings. In addition, the midwives’ ambivalence to the birthing rooms and the office could be an expression of powerful social norms in hospital labour wards that have been identified as appearing busy, while doing “busy work” (Davis & Homer, 2016), instead of being with the women in the birthing rooms (Hunter, 2004, 2002), which has also been described as “vigilant attendance” and “the art of doing ‘nothing’ well” (Kennedy, 2000). A birthing culture with an overly reductionist perspective could result in obstetrical mistreatment (Bohren et al., 2015). In high-income countries, this has been identified as being disrespectful and abusive treatment (Beck, 2018) in the form of poor relationships between women and care providers (Beck, 2018; Bohren et al., 2015) and the objectification and surveillance of women’s bodies (Bohren et al., 2015; Nilsson, 2014). Such experiences could have implications in women for a subsequent fear of childbirth, as well as post-traumatic stress syndrome (Ayers, Bond, Bertullies, & Wijma, 2016; Nilsson et al., 2018; Stenglin & Foureur, 2013).

In this study, relationships with women could be experienced as difficult when based on the women’s terms and attitudes that might not necessarily be in line with midwives’ expectations, as illustrated in the theme “Old and new caring roles of the midwife”. This relates to how the enhancement of quality relationships between midwives and women is important for effective care (Hunter et al., 2008; ICM, 2005) and how this links to the previously-mentioned QMNC framework of the global philosophy of optimising midwifery and normal biological, psychosocial and cultural childbirth processes (Renfrew et al., 2014).

It has been suggested that the presence of a midwife with a woman is crucial to the development of a different kind of midwifery knowledge and skills (Olafsdottir, 2006/2011) and that it impacts the midwife’s embodied and grounded knowledge in relation to each woman (Berg et al., 2012). It was noteworthy how the midwives in this study questioned whether they were practising real midwifery and woman-centred care in this labour ward. The reasoning for this could be that the reductionist paradigm is slowly changing the role of the midwife. Midwives spend less time with women during the birth process and instead spend time complying with organizational demands and the dominant medical model of care. This could leave them at risk of losing or not developing their grounded midwifery knowledge, i.e., theoretical, experience-based and intuitive knowledge in relation to the individual woman (Berg et al., 2012; Magistretti et al., 2016).

As part of the MiMo research project, this study was conducted before the MiMo (Berg et al., 2012) was introduced as an intervention in the research setting. It is interesting to note how the findings are in accordance with key themes of the MiMo, such as cultural norms and their influence on a midwifery approach to care and the balancing act of the midwife in meeting a woman’s needs and the (sometimes) challenging institutional guidelines that are based on a dominant medical approach. The reciprocal relationship—the presence and connections between midwives and women—were perceived by many as being pivotal during births, such as in the example above regarding techniques for helping some women with epidurals to become aware of their own body and its ability to give birth. The midwives also described their skills and strategies for creating a calm and trusting birthing atmosphere by promoting the normality of birth, which is also described in the MiMo. Furthermore, midwives were observed using different kinds of knowledge in relation to women’s individual needs (Berg et al., 2012). In this sense, the balancing act of the midwife and his/her grounded knowledge are important when dealing with contrasting models of care in the movement between the different birthing rooms and the office and with midwifery skills that do not form part of official clinical guidelines.

Preventing the midwives from acting as autonomous professionals appeared to create doubt and uncertainty regarding their own knowledge, regarding their caring role, and whether they were aware of the genuine needs of a woman during childbirth. This contrasts with how midwives feel more confident when working in birth centres or attending home births where they feel guided by a woman’s needs rather than hospital ward guidelines (Davis & Homer, 2016), and where they provide continuous supportive care that has positive outcomes on labour and birth (Bohren et al., 2017; Sandall et al., 2016). The MiMo should also allow contrasting models of care to operate side by side in line with the individual needs of a woman and her family (Berg et al., 2012), thus helping midwives decide when to provide a “being” or “doing” kind of care, or both, as required by a woman and her family based on both the situation and cultural aspects.

### Strengths and limitations

One strength of this study is that the first author is a midwife who is familiar with the context and the profession, suggesting that, in comparison with a complete outsider, she could read between the lines and also take a natural role in the ward and the birthing room and get to know the birthing woman and her partner and build confidence more quickly. However, being a researcher, teacher and midwife was sometimes challenging and demanded a high level of self-awareness about pre-understandings in order to avoid the risk of bias. Moreover, in relation to the fieldwork and the observations, such different roles could create uncertainty about the new roles that emerged (Collings & Gallinat, 2010). Initially, adjusting to the new roles with the other midwives being informants instead of colleagues could be difficult. Some of the midwives stated that they felt discomfort in being observed by a colleague who is also a teacher. However, the researcher discovered that such experiences could be overcome by explaining that her main focus was not on the individual midwife or on being judgmental, but on the phenomenon of midwifery care in general with an emphasis on understanding. The researcher also made an effort to assist the midwife and the birthing woman, for instance, by fetching items that were missing, taking part in conversations and acting in a reassuring manner, thus having a positive and relaxing effect on the midwife and the woman (Dellenborg, 2013). A further strength of this study was the intellectual collaboration between the co-authors and their miscellaneous perspectives as midwives with diverse experience of the profession and theoretical input (Gadamer, 1995/1960; Skott, 2013a). The close interaction between the first and the last author who, importantly, is not a midwife and therefore had an outsider’s perspective on the material, provided the opportunity to find alternative interpretations (Gadamer, 1995/1960; Morse, 2016). Thus, the last author also functioned as a support to the first author in developing her reflexive stance in relation to the field, thereby trying to avoid interpretations based on her preunderstandings. As previously mentioned, vital parts of the regular research meetings involved formulating and questioning emerging interpretations, as well as discussing the researchers’ potential preunderstandings.

In this ethnographic study, six midwives were observed during their shifts, either day, evening or night shifts in a hospital-based labour ward. Field notes were taken during the observations and informal talks and brief interviews were conducted with the midwives. The data also consisted of the researcher’s reflection notes (Roper & Shapira, 2000) and in-depth interviews with two of the observed midwives, as well as additional in-depth interviews with a further two midwives. Based on the observer’s data collection, gathered over a period of two months, as well as her previous knowledge of the ward, we consider the study to be a thorough ethnographic study. In research using ethnography, the aim is not to observe as many participants as possible but, through close reflexive participant observation, build confidence in order to be able to understand the participants in their context. Roper and Shapira (2000, p. 13) claim “(t)here seems to be no general rule … but there are reports of fieldwork that lasted years, and of fieldwork that lasted only a few months or weeks”.

Nevertheless, we need to bear in mind that there were also methodological limitations that should be considered when interpreting the results. First, being a midwife and, moreover, being part of introducing the MiMo, the observer might have created uncomfortable feelings of being controlled among her midwife colleagues. Yet, by addressing this dilemma in relation to the observed midwives, these feelings apparently disappeared. Secondly, being a sole observer and observing one ward only is a limitation. Thus, it would have been interesting to have been able to compare several delivery wards. Nevertheless, important experiences from this study might be transferred to and compared with other hospital-based labour wards (Sandall et al., 2016; World Health Organization, 2018) because the wider healthcare policies and hospital organization described here can be recognized in many healthcare settings in high-income countries.

## Conclusion

In the studied hospital-based labour ward, the midwives worked in a field of tension in which they had to balance contrasting models of childbirth care with ambivalent relationships between midwifery, medicine and the institutional demands for efficiency and bureaucracy. These circumstances obstructed the midwives’ freedom to act as autonomous professionals and created doubt and uncertainty regarding their own knowledge, regarding their caring role, and whether they were aware of the genuine needs of a woman during childbirth. The findings demonstrate the impact of the workplace culture on the content and meaning of midwives’ care, particularly their expectations and relationships with the birthing women that challenge the midwives’ caring roles in a new age of increased birth technology in obstetric-led hospital labour wards. This demonstrates the need for a woman-centred midwifery model to enhance and guide midwives’ care in the studied labour ward. Further research and implementation of a salutogenic, holistic model of care like the MiMo could, in practice, enhance the likelihood of the content and meaning of care being based on a midwifery approach, could make midwifery care more visible, and strengthen midwives’ autonomy, knowledge and skills, to the benefit of birthing women and their families. Given the strong evidence of improved outcomes of midwife-led care, such care in hospital labour wards should be promoted.

## Data Availability

The data that support the findings of this study are available on request from the corresponding author. The data are not publicly available due to restrictions, e.g., their containing information that could compromise the privacy of research participants.
